# Prevalence and genotyping of *Toxoplasma gondii* in naturally-infected synanthropic rats (*Rattus norvegicus*) and mice (*Mus musculus*) in eastern China

**DOI:** 10.1186/s13071-014-0591-6

**Published:** 2014-12-17

**Authors:** Chao Yan, Li-Jun Liang, Bei-Bei Zhang, Zhi-Long Lou, Hui-Feng Zhang, Xuan Shen, Yu-Qing Wu, Zi-Mu Wang, Ren-Xian Tang, Lin-Lin Fu, Kui-Yang Zheng

**Affiliations:** Department of Pathogenic Biology and Immunology, Laboratory of Infection and Immunity, Xuzhou Medical College, Xuzhou, Jiangsu Province 221004 PR China; Department of Clinical Medicine, Xuzhou Medical College, Xuzhou, Jiangsu Province 221004 PR China; State Key Laboratory of Veterinary Etiological Biology, Key Laboratory of Veterinary Parasitology of Gansu Province, Lanzhou Veterinary Research Institute, Chinese Academy of Agricultural Sciences, Lanzhou, Gansu Province 730046 PR China; School of Medical Technology, Xuzhou Medical College, Xuzhou, Jiangsu Province 221004 PR China

**Keywords:** Prevalence, *Toxoplasma gondii*, Genetic characterization, Synanthropic rodent, Eastern China

## Abstract

**Background:**

Synanthropic rats and mice share the same environment with humans and play an important role in epidemiology of toxoplasmosis; however, there is limited information about prevalence and genetic characterization of *Toxoplasma gondii* in synanthropic rats and mice in China.

**Findings:**

In the present study, the prevalence and genetic characterization of *T. gondii* naturally infected synanthropic rodents (*Rattus norvegicus* and *Mus musculus*) were investigated in the urban area of Xuzhou city, Eastern China between June 2013 and August 2014. DNA from the brain of each animal was prepared and screened by specific PCR assay targeting 35-fold repeated B1 gene (B1-PCR). PCR positive DNA samples were further genotyped by multi-locus PCR-RFLP. Overall, out of 123 synanthropic rodents, 29 samples were positive by B1 gene-targeted PCR (23.6%). Of these, 7 out of 31 (22.3%) *M. musculus* were positive, whereas the positive rate of *R. norvegicus* was 23.9% (22/92). Multi-locus PCR-RFLP analysis reveals that seven PCR-positive samples were completely genotyped and they were identified as type China 1 (ToxoDB# 9).

**Conclusion:**

To our knowledge, this is the first report of molecular detection and genetic characterization of *T. gondii* infection in synanthropic rodents in Eastern China. The results of the present study showed a high infection pressure of *T. gondii* exists in the environment and synanthropic rodents infected by *T. gondii* may be an important source of infection for cats and other animals.

## Background

Toxoplasmosis caused by the obligatory intracellular protozoan *Toxoplasma gondii* is a widespread zoonosis [1]. It is estimated that up to 30% of the human population of the world is suffering chronic infection with generally benign or mild nonspecific clinical symptoms [2]. Moreover, deaths and great morbidity can be brought about in fetuses and immunocompromised patients [3]. Humans and other animals can get infected mainly through consumption of undercooked meats containing cysts of *T. gondii* and ingestion of oocysts in environment. In addition, *T. gondii* can be also transmitted vertically from an infected mother to her baby during her first gestation [4].

Synanthropic rodents are widely distributed in China and it has been reported that the main species of rodents distributed in China may vary due to different climates, food sources and other factors [5]. However, brown rats (*Rattus norvegicus*) and synanthropic mice (*Mus musculus*) are widely distributed in the urban area of Noth China [6]. Naturally-infected rodents serving as important reservoir hosts play a key role in dissemination of *T. gondii* to other animals including cats since they are the main prey for cats and other stray carnivorous animals [7]. Furthermore, free-living animals such as stray cats and rodents could be used as sentinels of environmental spreading with *T. gondii* in densely built urban areas as they are exposed without any protection to all the infective forms of the parasite and feed on various sources of food on the ground [8-10].

Our previous study showed that there was a high prevalence of *T. gondii* in stray dogs and cats both in urban and rural areas of Xuzhou city, suggesting a high infection pressure for both animals and humans in that area [11]. However, the sources for this relative high prevalence of *T. gondii* in stray dogs and cats remain unclear. Moreover, knowledge about the prevalence and genetic characterization of *T. gondii* in synanthropic rodents in China is rather limited. Therefore, in the present study, we determined the prevalence of *T. gondii* in synanthropic rodents in Eastern China by detecting *T. gondii* DNA using specific PCR targeting 35-fold repeated B1 gene (B1-PCR), and genotyped *T. gondii* in synanthropic rodents.

## Findings

### Materials and methods

#### Sample collection and preparation

A total of 123 rodents were randomly collected from Tongshan District, Yunlong District, GuLou District and Peixian County in Xuzhou City, Jiangsu Province, Eastern China during July 2013 to August 2014. The geographical information of Xuzhou City was described in detail elsewhere [11]. Animals were trapped and transported to our laboratory where the animals were anaesthetized and whole brain from each animal was obtained and stored at −20°C until use. The age of animals was estimated by body’s length as description elsewhere [6], and these animals were divided into four groups according to their ages: Juvenile group (with the body length < = 110 mm), Sub-adult group (with the body length 111–150 mm), adult group (with the body length 151–175 mm) and old group (with the body length >175 mm).

### DNA extraction and specific polymerase chain reaction

DNA extraction was performed using a commercial DNA extraction kit (Shanghai sangon biotech, Shanghai, China) according to manufacturer’s recommendations. Briefly, about 50 mg of each brain tissue was cut into small pieces, homogenized in 200 μl of DNA extraction buffer and proteinase K, and added for ingestion at 55°C for 4 h. Subsequently, 500 μl buffered phenol was added and centrifuged at 12,000 g for 5 min. DNA was extracted twice using phenol-chloroform, and stored at −20°C until use after precipitation by sodium acetate and ethanol.

To estimate the prevalence of *T. gondii* in synanthropic rodents in Eastern China, specific PCR that targets 35-fold repeated B1 gene (B1-PCR) was employed to detect the possible infection with *T. gondii* in synanthropic rats and mice [12,13]. Positive control of DNA from *T. gondii* infected mice experimentally and negative controls were included in each test.

### Genetic characterization of *T. gondii* in positive DNA samples

Genetic characterization of *T. gondii* in randomly selected positive DNA samples in synanthropic rodents were performed using the multilocus PCR-RFLP method [14-17]. Briefly, a total of 10 genetic markers (i.e., SAG1, SAG2, alter.SAG2, SAG3, BTUB, GRA6, c22-8, L358, PK1, and Apico) were amplified by multiplex PCR using external primers. The PCR reaction (25 μl) consisting of 1 × PCR buffer, 0.2 mM of each primer, 200 μM dNTPs, 2 mM MgCl_2_, 0.2 U of HotStart Taq DNA polymerase (TAKARA, Japan) were carried out using a thermal cycler (PTC 200, Bio-RAD) under the reaction conditions as follows: 95°C for 5 min to activate the DNA polymerase, then 30 cycles of PCR at 95°C for 30 s, 55°C for 60 s and 72°C for 90 s. Six references including GT1, PTG, CTG, MAS, TgCgCa1 and TgCatBr5 were employed as positive controls in each reaction. The amplified DNA fragments were diluted 1:1 in sterile, double-distilled water and then were amplified by internal primers for each locus, respectively [14-17]. A similar approach was used for nest PCR, the annealing temperature of which is at 60°C for 60 s for all the markers except Apico (55°C for 60 s). The nest PCR amplified products were then digested with restriction enzymes for 1 h at recommended temperature according to the instructions for each enzyme. The digested fragments were resolved in 2.5%-3% agarose gel and visualized using a gel documentation system (UVP GelDoc-ItTM Imaging System, Cambridge, U.K.).

### Statistical analysis

Differences in the prevalence of *T. gondii*-infected rats and mice among different variables including location, age and gender were analyzed using a Chi-square test by SPPS (Release 16.0 standard version, SPPS Inc., Chicago, America). Statistical differences were found when *P* < 0.05.

## Results and discussion

*T. gondii* DNA in brains was demonstrated in 29 out of 123 (23.6%) rodents collected. Of these, 7 out of 31 (22.6%) mice were *T. gondii* DNA-positive whereas the prevalence of *T. gondii* in rats was 23.9% (22 out of 92 animals examined, Table 1). There was no statisticaldifference in *T. gondii* prevalence in species, genders and regions where samples were collected. However, statistically significant difference was found in the prevalence of *T. gondii* in synanthropic rodents of different ages (*P* < 0.01), in which the prevalence of *T. gondii* in old group (75.0%) and juvenile group (23.1%) were significantly higher than that of sub-adult group (19.0%) and adult group (19.2%), respectively (Table 1), suggesting that both congenital infection and acquired infection of *T. gondii* existed in naturally infected synanthropic rodents in this area.

**Table 1:** **The prevalence of** ***Toxoplasma gondii*** **infection in synanthropic rats and mice in Xuzhou City, Eastern China**

**Variable**	**No. examined**	**No. positive**	**Prevalence (%)**
Region			
Tongshan District	26	6	23.1
GuLou District	83	21	25.3
Other areas	14	2	14.3
Gender			
Female	78	18	23.1
Male	45	11	24.4
Age*			
Juvenile group	26	6	23.1
Sub-adults group	63	12	19.0
Adults group	26	5	19.2
Old group	8	6	75.0
Species			
*Mus musculus*	31	7	22.6
*Rattus norvegicus*	92	22	23.9
Total	123	29	23.6

*Means P < 0.01 when variables were analyzed using a Chi-square test.

DNA samples were selected for genetic characterization using multilocus PCR-RFLP. Multilocus PCR-RFLP results showed that only seven DNA samples showed complete genotyping results and the genotypes were identified as ToxoDB #9 (China 1, Table 2). Unfortunately, other DNA samples showed no results or part genotype results, thus the genotypes could not be determined.

**Table 2:** Summary of genotyping of *Toxoplasma gondii* in synanthropic rats and mice in China

**Isolate ID**	**Host**	**Tissue**	**Location**	**SAG1**	**5' + 3’ SAG2**	**Alternative SAG2**	**SAG3**	**BTUB**	**GRA6**	**c22-8**	**c29-2**	**L358**	**PK1**	**Apico**	**Genotype**
GT1	Goat		United States	I	I	I	I	I	I	I	I	I	I	I	Reference, Type I, ToxoDB #10
PTG	Sheep		United States	II/III	II	II	II	II	II	II	II	II	II	II	Reference, Type II, ToxoDB #1
CTG	Cat		United States	II/III	III	III	III	III	III	III	III	III	III	III	Reference, Type III, ToxoDB #2
MAS	Human		France	u-1*	I	II	III	III	III	u-1*	I	I	III	I	Reference, ToxoDB #17
TgCgCa1	Cougar		Canada	I	II	II	III	II	II	II	u-1*	I	u-2*	I	Reference, ToxoDB #66
TgCatBr5	Cat		Brazil	I	III	III	III	III	III	I	I	I	u-1*	I	Reference, ToxoDB #19
TgWtdSc40	WTD		USA	u-1	II	II	II	II	II	II	II	I	II	I	Type 12, ToxoDB #5
TgCatBr64	Cat		Brazil	I	I	u-1	III	III	III	u-1	I	III	III	I	Reference, ToxoDB #111
TgRsCr1	Toucan		Costa Rica	u-1	I	II	III	I	III	u-2	I	I	III	I	Reference, ToxoDB #52
TgRn05	Rat	Brain	Xuzhou, China	u-1	II	II	III	III	II	II	III	II	II	I	ToxoDB #9
Tgmouse07	Mouse	Brain	Xuzhou, China	u-1	II	II	III	III	II	II	III	II	II	I	ToxoDB #9
Tgmouse08	Mouse	Brain	Xuzhou, China	u-1	II	II	III	III	II	II	III	II	II	I	ToxoDB #9
TgRn13	Rat	Brain	Xuzhou, China	u-1	II	II	III	III	II	II	III	II	II	I	ToxoDB #9
TgRn14	Rat	Brain	Xuzhou, China	u-1	II	II	III	III	II	II	III	II	II	I	ToxoDB #9
TgRn15	Rat	Brain	Xuzhou, China	u-1	II	II	III	III	II	II	III	II	II	I	ToxoDB #9
Tgmouse23	Mouse	Brain	Xuzhou, China	u-1	II	II	III	III	II	II	III	II	II	I	ToxoDB #9

*u-1 and u-2 represent unique RFLP genotypes, respectively.

WTD: White-tailed Deer.

nd: not determined.

Synanthropic rodents which share the same environment with humans are considered as an important reservoir of *T. gondii* for cats and other animals because naturally infected rodents constitute important prey for wild and synanthropic felids [18]. However, there is limited information about prevalence of *T. gondii* in synanthropic rodents in China [19]. In addition, the relative high prevalence of *T. gondii* in synanthropic rodents in the present study might partly account for the high infection rates of *T. gondii* in cats and dogs in the same area [11]. However, PCR assay can only provide a suggestive, but not conclusive evidence for *T. gondii* infection in rodents. Therefore, our results in the present study showed the preliminary but fundamental data for further studies which aim at isolating live parasites in synanthropic rodents.

The information of genetic characterization of *T. gondii* in synanthropic rodents is rather limited [20]. The genotypes identified in this study were the genotype ToxoDB #9, which is dominantly prevalent in cats and other animals in most parts of China [21,22]. More importantly, this genotype which was also identified in naturally infected cats in this region (Eastern China) showed a moderate or high virulence to mice, indicating that the circulating *T. gondii* in cats and synanthropic rodents could cause severe toxoplasmosis in humans if it were to spread to humans [22]. Surprisingly, ToxDB #10 and ToxDB #205 which were also prevalent in naturally-infected cats in this region were absent in our study, suggesting that there might be additional sources for cats infected by *T. gondii* [23]*.*

## Conclusions

This is the first report of molecular detection and genetic characterization of *T. gondii* in synanthropic rodents in Eastern China. The results of the present study revealed a wide distribution of *T. gondii* in synanthropic rats and mice in China and an identical genotype circulating in rodents in this region, which provide basic information for further prevention and control of toxoplasmosis in humans.

## References

[1] Dubey JP (2010). Toxoplasmosis of Animals and Humans.

[2] Tenter AM, Heckeroth AR, Weiss LM (2000). *Toxoplasma gondii*: from animals to humans. Int J Parasitol.

[3] Sibley LD, Boothroyd JC (1992). Virulent strains of *Toxoplasma gondii* comprise a single clonal lineage. Nature.

[4] Montoya JG, Liesenfeld O (2004). Toxoplasmosis. Lancet.

[5] Zhou P, Chen Z, Li HL, Zheng H, He S, Lin RQ, Zhu XQ (2011). *Toxoplasma gondii* infection in humans in China. Parasit Vectors.

[6] Zheng ZM, Jiang ZK, Chen AG (2008). Rodent Zoology.

[7] Dubey JP, Frenkel JK (1998). Toxoplasmosis of rats: a review, with considerations of their value as an animal model and their possible role in epidemiology. Vet Parasitol.

[8] Hughes JM, Williams RH, Morley EK, Cook DA, Terry RS, Murphy RG, Smith JE, Hide G (2006). The prevalence of *Neospora caninum* and co-infection with *Toxoplasma gondii* by PCR analysis in naturally occurring mammal populations. Parasitology.

[9] Meerburg BG, De Craeye S, Dierick K, Kijlstra A (2012). *Neospora caninum* and *Toxoplasma gondii* in brain tissue of feral rodents and insectivores caught on farms in the Netherlands. Vet Parasitol.

[10] Meireles LR, Galisteo AJ, Pompeu E, Andrade HF (2004). *Toxoplasma gondii* spreading in an urban area evaluated by seroprevalence in free-living cats and dogs. Trop Med Int Health.

[11] Yan C, Fu LL, Yue CL, Tang RX, Liu YS, Lv L, Shi N, Zeng P, Zhang P, Wang DH, Zhou DH, Zhu XQ, Zheng KY (2012). Stray dogs as indicators of *Toxoplasma gondii* distributed in the environment the first report across an urban–rural gradient in China. Parasit Vectors.

[12] Hill DE, Chirukandoth S, Dubey JP, Lunney JK, Gamble HR (2006). Comparison of detection methods for *Toxoplasma gondii* in naturally and experimentally infected swine. Vet Parasitol.

[13] Homan WL, Vercammen M, De Braekeleer J, Verschueren H (2000). Identification of a 200- to 300-fold repetitive 529 bp DNA fragment in *Toxoplasma gondii,* and its use for diagnostic and quantitative PCR. Int J Parasitol.

[14] Tian YM, Huang SY, Miao Q, Jiang HH, Yang JF, Su C, Zhu XQ, Zou FC (2014). Genetic characterization of *Toxoplasma gondii* from cats in Yunnan Province, Southwestern China. Parasit Vectors.

[15] Jiang HH, Huang SY, Zhou DH, Zhang XX, Su C, Deng SZ, Zhu XQ (2013). Genetic characterization of *Toxoplasma gondii* from pigs from different localities in China by PCR-RFLP. Parasit Vectors.

[16] Zhang XX, Lou ZZ, Huang SY, Zhou DH, Jia WZ, Su C, Zhu XQ (2013). Genetic characterization of *Toxoplasma gondii* from Qinghai vole, Plateau pika and Tibetan ground-tit on the Qinghai-Tibet Plateau, China. Parasit Vectors.

[17] Chen J, Li ZY, Zhou DH, Liu GH, Zhu XQ (2012). Genetic diversity among *Toxoplasma gondii* strains from different hosts and geographical regions revealed by sequence analysis of GRA5 gene. Parasit Vectors.

[18] Murphy RG, Williams RH, Hughes JM, Hide G, Ford NJ, Oldbury DJ (2008). The urban house mouse (*Mus domesticus*) as a reservoir of infection for the human parasite *Toxoplasma gondii*: an unrecognised public health issue?. Int J Environ Health Res.

[19] Yin CC, He Y, Zhou DH, Yan C, He XH, Wu SM, Zhou Y, Yuan ZG, Lin RQ, Zhu XQ (2010). Seroprevalence of *Toxoplasma gondii* in Rats in Southern China. J Parasitol.

[20] Wang L, Cheng HW, Huang KQ, Xu YH, Li YN, Du J, Yu L, Luo QL, Wei W, Jiang L, Shen JL (2013). *Toxoplasma gondii* prevalence in food animals and rodents in different regions of China: isolation, genotyping and mouse pathogenicity. Parasit Vectors.

[21] Dubey JP, Zhu XQ, Sundar N, Zhang H, Kwok OC, Su C (2007). Genetic and biologic characterization of *Toxoplasma gondii* isolates of cats from China. Vet Parasitol.

[22] Zhou P, Zhang H, Lin RQ, Zhang DL, Song HQ, Su C, Zhu XQ (2009). Genetic characterization of *Toxoplasma gondii* isolates from China. Parasitol Int.

[23] Wang L, Chen H, Liu D, Huo X, Gao J, Song X, Xu X, Huang K, Liu W, Wang Y, Lu F, Lun ZR, Luo Q, Wang X, Shen J (2013). Genotypes and mouse virulence of *Toxoplasma gondii* isolates from animals and humans in China. PLoS One.

